# The understanding of the impact of efficiently optimized underlap length on analog/RF performance parameters of GNR-FETs

**DOI:** 10.1038/s41598-023-40711-7

**Published:** 2023-08-24

**Authors:** Md Akram Ahmad, Jitendra Kumar

**Affiliations:** Department of Electronics Engineering, IIT Dhanbad, Dhanbad, 826004 India

**Keywords:** Materials science, Nanoscience and technology

## Abstract

The aim of this study is to examine the analog/RF performance characteristics of graphene nanoribbon (GNR) field-effect transistors (FETs) using a novel technique called underlap engineering. The study employs self-consistent atomistic simulations and the non-equilibrium Green's function (NEGF) formalism. Initially, the optimal underlap length for the GNR-FET by device has been determined evaluating the ON-current (*I*_*ON*_) to OFF-current (*I*_*OFF*_) ratio, which is a critical parameter for digital applications. Subsequently, the impact of underlap engineering on analog/RF performance metrics has been analyzed and conducting a comprehensive trade-off analysis considering parameters such as intrinsic-gain, transistor efficiency, and device cut-off frequency. The results demonstrate that the device incorporating the underlap mechanism exhibits superior performance in terms of the *I*_*ON*_*/I*_*OFF*_ ratio, transconductance generation factor (TGF), output resistance (*r*_*0*_), intrinsic gain (*g*_*m*_*r*_*0*_), gain frequency product (GFP), and gain transfer frequency product (GTFP). However, the device without the underlap effect demonstrates the highest transconductance (*g*_*m*_) and cut-off frequency (*f*_*T*_). Finally, a linearity analysis has been conducted to compare the optimized GNR-FET device with the conventional GNR-FET device without the underlap effect.

## Introduction

In recent decades, there has been a notable decrease in the size of transistors, moving from micrometers to nanometers, driven by the well-known Moore’s Law^1,2^. However, as the demand for advanced electronic devices continues to rise, the size limitations of silicon-based transistors have become increasingly challenging, and there will eventually be physical limits to further miniaturization. The main obstacle in this regard is the occurrence of short-channel effects (SCE), such as leakage current, subthreshold swing (SS), drain-induced barrier lowering (DIBL), and velocity saturation, which are consequences of decreasing the distance between the source and drain^3–5^. In recent times, researchers have actively pursued extensive research to explore novel materials that could overcome these limitations. Subsequently, graphene has emerged as a highly significant material that has captured significant attention in the field of electronic devices. This is primarily due to its abundant availability and cost-effective attributes, making it an exceptionally attractive option for various electronic applications^6^.

Graphene, consisting of a single layer of carbon atoms, has positioned itself as an exceptionally promising material for future semiconductor devices, especially in high-frequency applications. This is primarily attributed to its remarkable properties, including outstanding thermal conductivity, high saturation velocity, flexibility, impressive mechanical strength, and superior carrier mobility^7–11^. Moreover, graphene's exceptional mobility characteristics make it an excellent candidate for flexible and radio frequency (RF) device applications^12,13^. In addition to its advantageous characteristics, at relatively short channels, the lack of band gaps in graphene results poor current ON/OFF ratio (I_ON_/I_OFF_). Thus, graphene nanoribbon (GNR) needs to be made to use graphene as a device, and the device based on graphene is known as a graphene nanoribbon (GNR) field-effect transistor (FET)^14,15^.

Various approaches have been explored to enhance the electrical performance of GNR-based FETs. These methods include utilizing different gate-oxide dielectric materials, channel doping, dimensional scaling, selecting gate materials with specific work functions, and introducing vacancy defects on the channel^16–25^. However, there is still significant room for investigation, particularly in the area of channel-length engineering. Previous studies have demonstrated that implementing a gate-underlap structure can improve leakage current, subthreshold swing (SS), and current ON/OFF ratio^26^. The introduction of underlap architectures helps in reducing short-channel effects (SCEs) by adjusting the effective channel length of the device^27^. It also mitigates fringing capacitance^28^ and Gate Induced Drain Leakage (GIDL)^29^, resulting in reduced switching power and improved suitability for logic applications. However, the underlap between the gate and the source or drain leads to an increase in channel resistance, which diminishes the ON-current and adversely affects device performance. To address this issue, an asymmetric underlap structure, where the underlap is applied on the drain side, is preferred^30^. Despite these advancements, the existing methods for enhancing the analog/RF performance of FETs remain inadequate. As a result, recent studies have focused on improving the analog/RF performance of GNR-FETs. This motivates further investigation into the analog and RF performance characteristics of GNR-FETs with underlap structures. Notably, there is a lack of prior research examining the analog/RF performance behavior of GNR-FETs employing the asymmetric underlap mechanism.

This research study focuses on examining the impact of underlap engineering on analog/RF parameters in GNR-FETs for low-power applications. To achieve this objective, the non-equilibrium Green's function (NEGF) methodology is employed to investigate the figure-of-merits (FOMs) related to analog and RF performance in GNR-FET devices with varying underlap lengths. Key parameters such as the gain frequency product (GFP) and gain transfer frequency product (GTFP) are analyzed, as they are crucial for circuit design and high-speed switching applications. The findings of this study can serve as a valuable resource for researchers involved in the design of novel GNR-FETs that exhibit superior performance compared to conventional FETs. Moreover, it is expected that this research will inspire further exploration of the application potential of GNR-FETs in diverse multidimensional contexts.

### Device structure and simulation methodology

**Figure 1:**
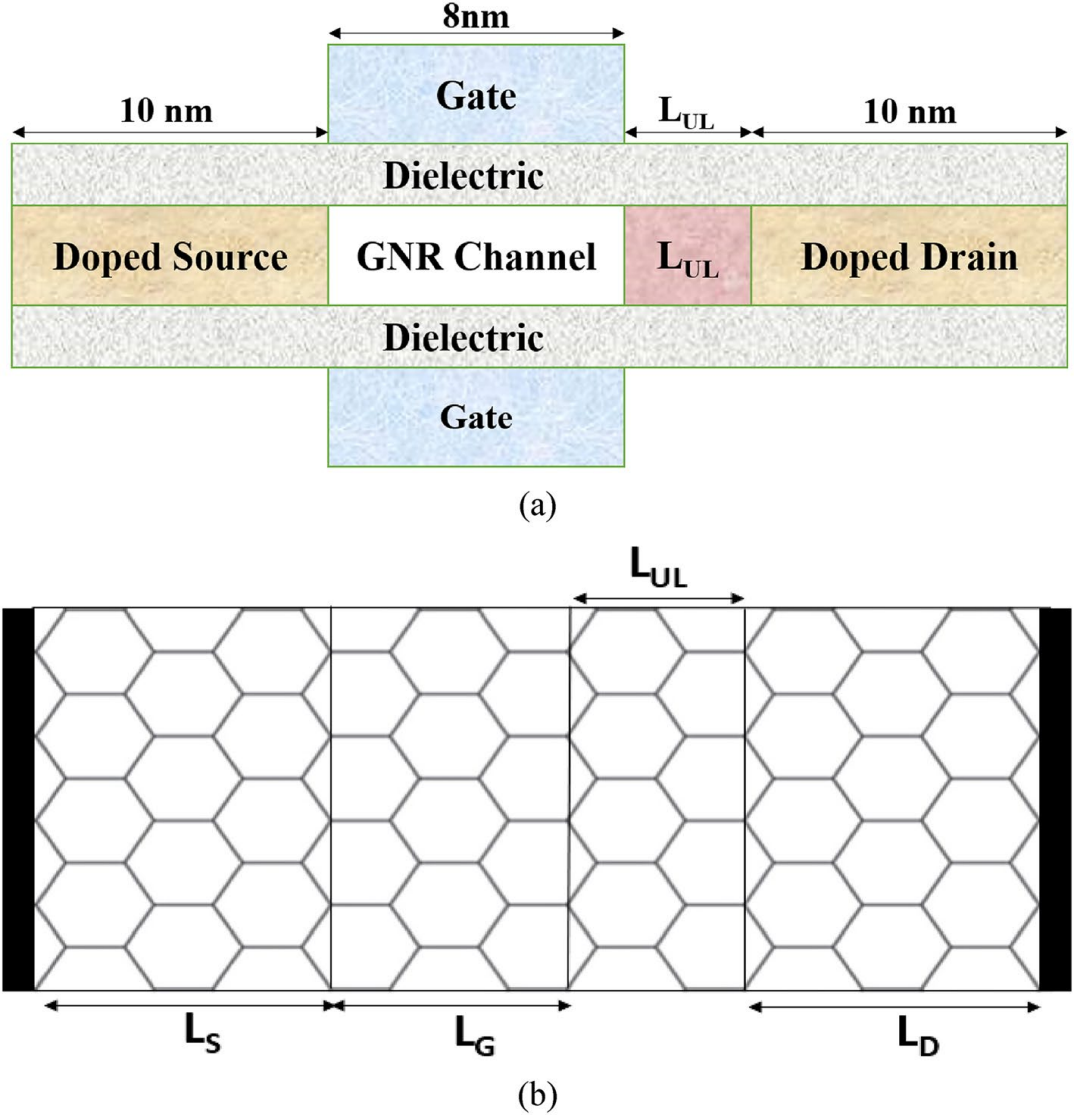
Schematic of GNR-FET: (**a**) Cross-sectional view (**b**) Top view (The image only shows the surface of GNR).

Figure 1a and b illustrate the cross-sectional view and top view, respectively, of the simulated 12-armchair double-gated (DG) GNR-FET with underlap engineering. The channel and the source/drain are formed by a 1.37 nm-wide 2D graphene sheet. The lattice constant in GNR is 2.46 Å, and the carbon–carbon (C–C) bond length (d) is 1.42 Å. Our focus on 12-armchair GNRs stems from previous research suggesting that a bandgap of 0.6 eV and an effective mass of 0.064 *m*_*0*_^31–33^, where *m*_*0*_ represents the free mass of an electron, are essential for achieving optimal performance. The top and bottom gate oxide layers are composed of *HfO*_*2*_. We vary the underlap length from 0-nm to 10-nm, incrementing by 2-nm. The source and drain regions are doped with n-type dopants, while the underlap and channel length (*L*_*G*_ = 8 nm)^26^ are intrinsic regions. The simulations are conducted with a fixed drain-to-source voltage (*V*_*DS*_) at a temperature of 300 K. The parameters used in the device simulation are presented in Table 1.

**Table 1:** Parameters of the GNR-FET structure.

Symbols	Definition	Nominal values/range of values
L_G_	Gate length	8 nm
L_S_	Source length	10 nm
L_D_	Drain length	10 nm
L_UL_	Underlap length	(0–10) nm
t_ox_	Oxide thickness	2 nm
K	Dielectric constant	16
N_D_	Source and Drain doping	2.5 × 10^13^ cm^−2^
N_C_	Channel doping	0
E_G_	Gap Energy	0.6 eV
m*	Effective mass	0.064 *m*_*0*_
V_DS_	Drain-source voltage	0.3 V

To accomplish the objective, the NanoTCAD ViDES atomistic device simulator perform all simulations within the non-equilibrium Green's function (NEGF) framework^34^. The tight-binding approximation is used to describe the interactions between individual carbon atoms in a graphene nanoribbon (GNR) at an atomic level. These interactions specifically involve the C–C atoms and are limited to the nearest neighboring atoms. In the NEGF approach, first-of-all, an appropriate Hamiltonian matrix for the channel is taken into account. The simulation employed a 2-band Hamiltonian, expressed as follows^35,36^:

1 $$H\left(k\right)=\left[\begin{array}{cc}{E}_{B}& tf(k)\\ {tf(k)}^{*}& {E}_{A}\end{array}\right],$$

the parameters E_A_ and E_B_ represent the energy levels at the top of the valence band and the bottom of the conduction band, respectively. These can be expressed as E_B_—E_A_ = E_G_, where E_G_ is the bandgap. Here, only one atomic orbital and the primitive unit cell comprises only one atom is considered, which leads to the formation of a single energy band. In this simulation, the in-plane hopping parameter, denoted as t, has a value of 2.7 eV^31^.

2 $$f\left(k\right)={e}^{j{k}_{y}d}+{e}^{-j{k}_{y}d/2}\mathrm{cos}\left(\sqrt{3}{k}_{x}d/2\right).$$

After defining the Hamiltonian matrix, Green's function is calculated as Ref.^37^:

3 $$ G\left( E \right) = \left[ {EI - H - \sum_{S} - \sum_{D} } \right]^{ - 1} , $$

which is examined by referencing earlier work^38^. After performing the Green's function calculation, the Schrödinger equation is solved with an open boundary condition to obtain the electron and hole concentrations. Subsequently, the electron density is calculated using Newton–Raphson iteration method. Ultimately, the Landauer formula^37^ is employed to compute the drain current (*I*_*D*_). In Green’s function equation, *E*, *I*, *H*, $\sum_{D}$ and $\sum_{S}$ represent energy, identity matrix, material Hamiltonian, and self-energy matrix at drain and source terminals, respectively.

## Results and discussion

**Figure 2:**
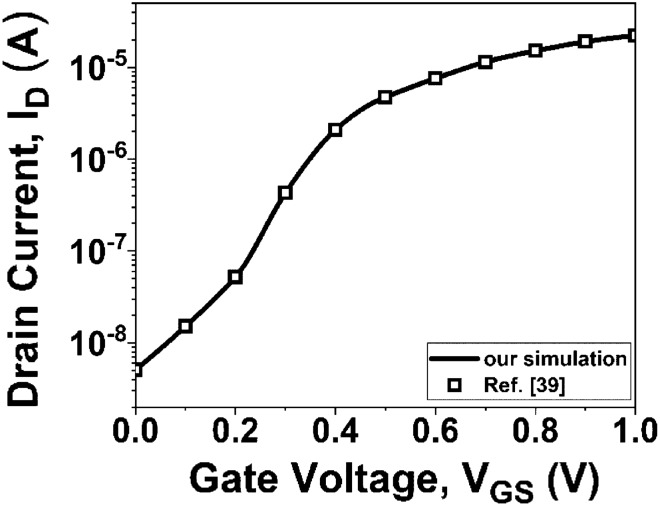
Calibration of *I*_*D*_*-V*_*GS*_ characteristics of the simulator and reported^39^ data.

To begin the analysis, the current simulator is calibrated to correspond to the device structure presented in Ref.^39^. Figure 2 demonstrates that obtained simulations are in good agreement with previous research findings.

Once the correctness of the simulation with the above-described methodology has been established, an underlap is introduced in the body of the GNR-FET at the drain end to assess the impact of Asymmetric underlap (UL) length on the performance of GNR-FET devices. It is important to clarify that whenever UL is mentioned unless explicitly stated otherwise, it refers to the default Asymmetric UL of drain extension.

### Optimization of underlap length

**Figure 3:**
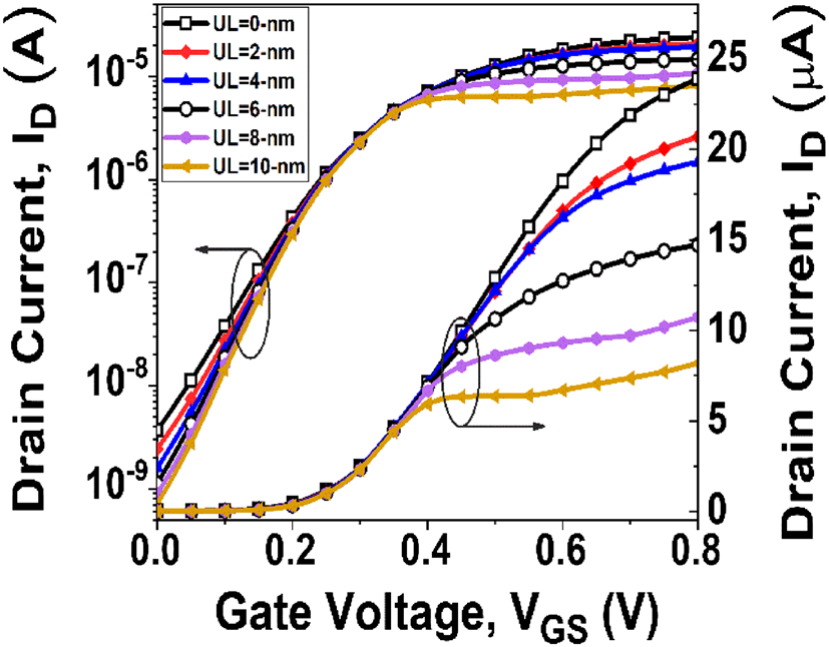
Transfer characteristics of GNR-FETs with various underlap length at *V*_*DS*_ = 0.3 V.

After confirming the accuracy, the impact of UL engineering on the transfer characteristics of GNR-FETs is examined. Figure 3 depicts the impact of UL length in the *I*_*D*_ as a function of gate-to-source voltage (*V*_*GS*_) of GNR-FETs in which the underlap length is varied from 0-nm to 10-nm with a step-size of 2-nm to get an optimized underlap state. The optimized state of the device is achieved by utilizing digital performance FOM, ON-current to OFF-current ratio. The *I*_*D*_ values for the ON-state and OFF-state are examined at *V*_*DS*_ = 0.3 V, *V*_*GS*_ = 0.8 V, and *V*_*DS*_ = 0.3 V, *V*_*GS*_ = 0 V, respectively.

**Figure 4:**
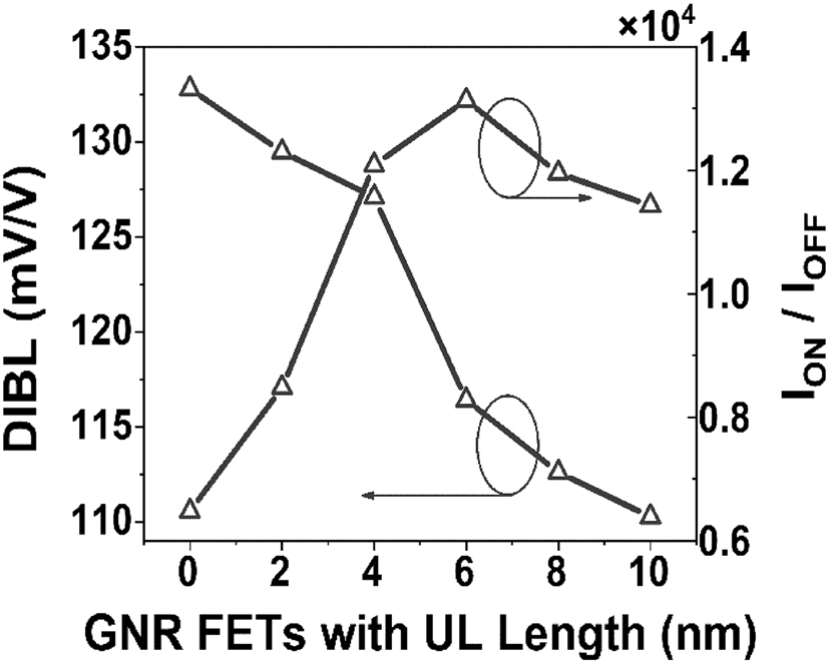
Variation of DIBL and current ON/OFF ratio for different underlap length.

**Figure 5:**
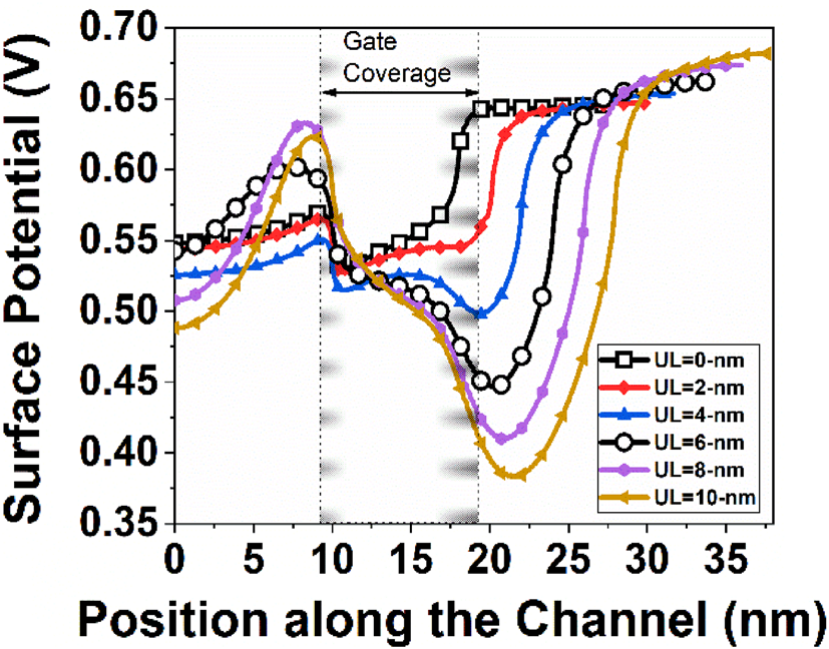
Surface potential plot of the device at *V*_*GS*_ = 0.8 V.

**Figure 6:**
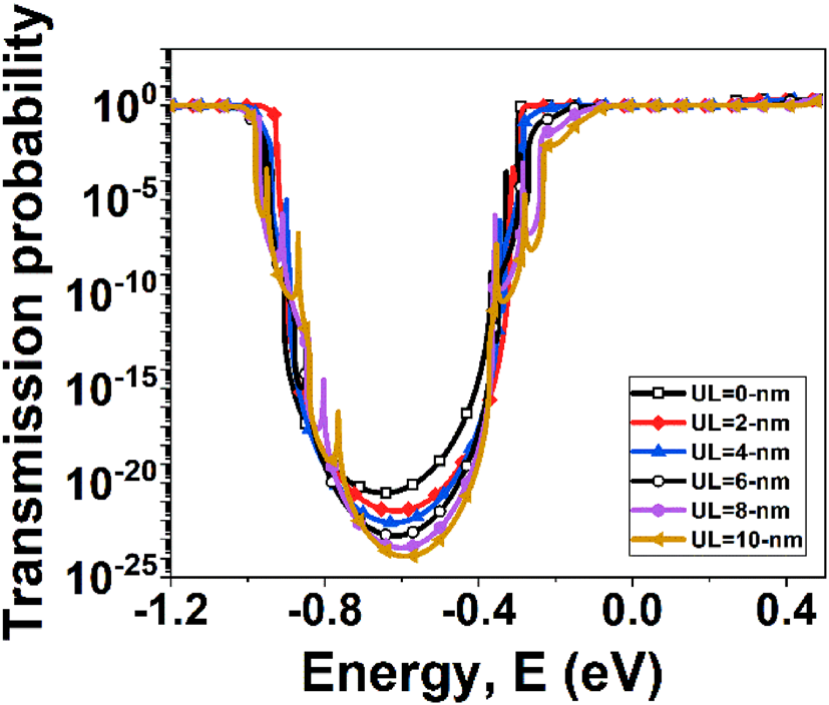
Transmission probability variation with energy at *V*_*GS*_ = 0.8 V.

It is observed from Fig. 4 that a significant amount of DIBL is present in the device with low underlap length, which implies a larger *I*_*OFF*_. When the underlap length increases, the DIBL decreases, resulting in a reduced *I*_*OFF*_ without considerably lowering *I*_*ON*_. As a result, the *I*_*ON*_*/I*_*OFF*_ ratio increases. The Underlap length eventually rises to a level where DIBL is no longer important. As a result, a small change in the *I*_*OFF*_ is seen as underlap increases. However, raising the underlap raises the total resistance of the channel, which dramatically reduces the *I*_*ON*_ after some point. As a result, *I*_*ON*_*/I*_*OFF*_ begins to decline. Figure 5 depicts the surface potential plot of the simulated structure for various underlap lengths. It is observed from Fig. 5 that the induced inversion charge of the device rises as the underlap length of the device increases. As a result, the potential barrier in the UL region is enhanced. Following the observation of the surface potential curve, the effects of UL engineering on the transmission window for carrier transmission are investigated. Figure 6 depicts the transmission probability variation with energy, and it is observed that with the increase of UL length of the device, the transmission probability curve decreases, which results smaller drain current^39^.

From the above graphs and discussion, it is clear that the optimal underlap point is achieved at a UL length of 6-nm. Henceforth, an UL length of up to 6-nm is elected for further analysis and compare the results with a conventional GNR-FET device having zero underlap gap between the gate and drain regions.

### Analog performance

The analog performance FOMs of the GNR-FET device are discussed in this section. The parameters investigated and analyzed here are as follows: the transconductance (*g*_*m*_), transconductance generation factor (TGF), output resistance (*r*_*0*_), and intrinsic gain (*A*_*V*_). The parameters *g*_*m*_ and TGF are expressed as follows:

4 $${g}_{m}= \frac{\partial {I}_{D}}{\partial {V}_{GS}}$$

5 $$TGF=\frac{{g}_{m}}{{I}_{D}}$$

**Figure 7:**
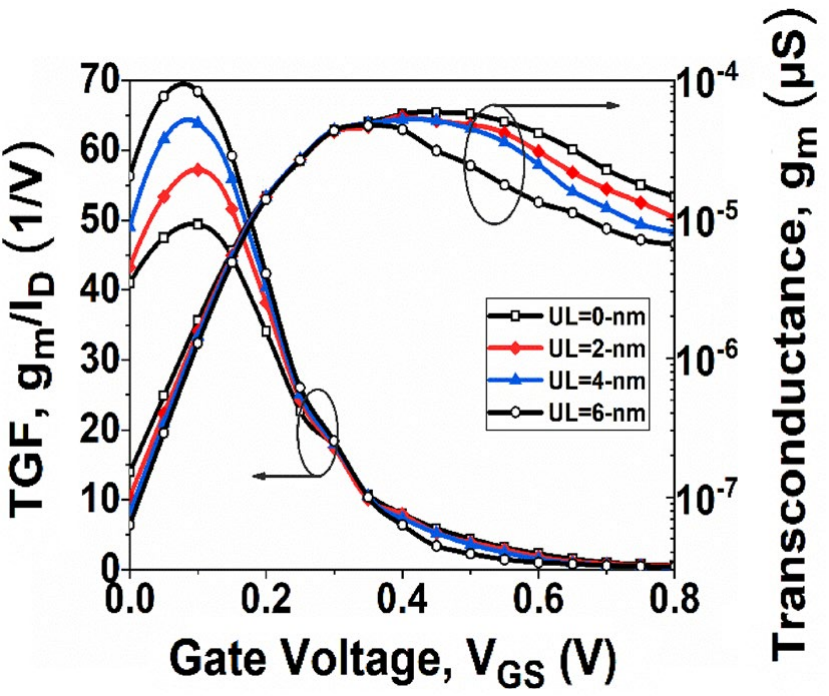
Variation of *g*_*m*_ and TGF with *V*_*GS*_ at *V*_*DS*_ = 0.3 V.

Figure 7 depicts the changes in *g*_*m*_ concerning* V*_*GS*_, where it is observed that initially *g*_*m*_ increases rapidly with gate voltage and finally appears to peak and then decreases. This rising and falling tendency in *g*_*m*_ is due to the *I*_*D*_ variation of the device with *V*_*GS*_. It is evident that the device with UL = 6-nm has a lower *g*_*m*_. It is because of degraded mobility in the channel due to increased channel resistance with UL engineering. The TGF is another crucial factor for analog applications. The concept of TGF refers to the effective utilization of drain current in achieving a desirable *g*_*m*_ value. A higher TGF value suggests that the device is well-suited for low-power amplifier designs. Figure 7 depicts the variation of TGF with respect to *V*_*GS*_. It is observed from Fig. 7 that the TGF curve improves with UL structure at low *V*_*GS*_, although there is no substantial improvement with high *V*_*GS*_. Moreover, the maximum value of TGF is obtained with the UL = 6-nm structure due to the lower *I*_*D*_ in the GNR-FET with the UL effect.

The intrinsic gain (*A*_*V*_) is another significant FOM for analog operation. The *A*_*V*_ should be as high as possible for optimal analog performance. The *A*_*V*_ can be defined and calculated as follows:

6 $${A}_{V}= {g}_{m}{r}_{0}$$

**Figure 8:**
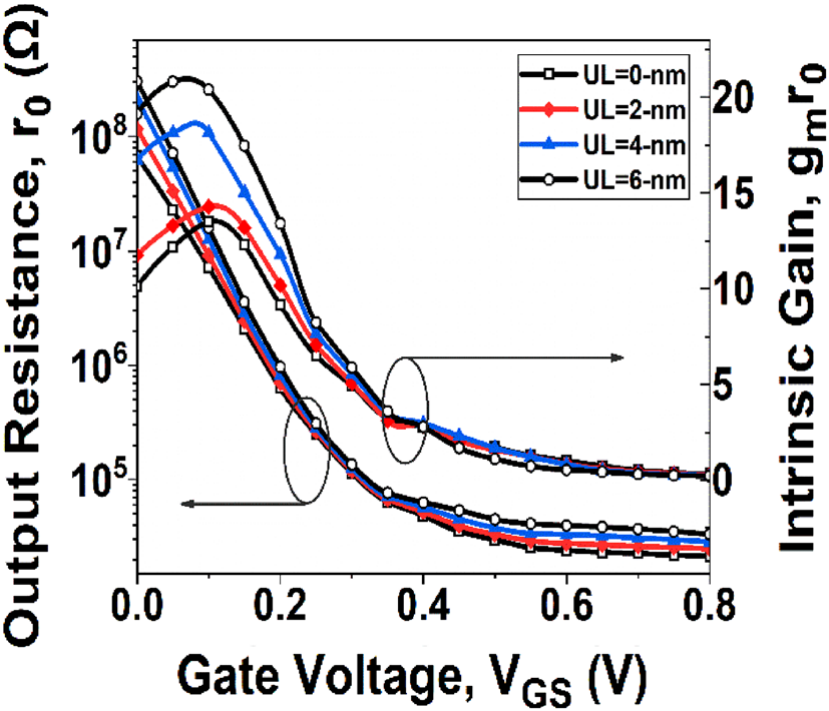
Plot of *r*_*0*_ and *A*_*V*_ with *V*_*GS*_ at *V*_*DS*_ = 0.3 V.

It is clear from the above equation that *A*_*V*_ depends on the device’s *r*_*0*_ and *g*_*m*_. Thus, understanding the variation of *r*_*0*_ is required before studying *A*_*V*_. It is observed from Fig. 8 that *r*_*0*_ increases with UL engineering. This is due to the enlargement in channel resistance with underlap length. As the channel resistance increases, the conductivity of the channel decreases. As a result, *r*_*0*_ increases with underlap length.

Figure 8 depicts the effect of underlap engineering on *A*_*V*_. It is evident from Fig. 8 that initially *A*_*V*_ increases, eventually appears at peak value, and then decreases. The initial rise in *A*_*V*_ can be attributed to the dominance of *g*_*m*_ over *r*_*0*_. As the gate voltage increases, the value of *g*_*m*_ approaches a constant value for shorter period of *V*_*GS*_, after which it decreases, while *r*_*0*_ keeps decreasing, leading to a Bell-shaped *A*_*V*_ curve.

### RF performance

To evaluate the effectiveness and feasibility of underlap on RF applications of devices, two essential RF FOMs; gate capacitance (*C*_*G*_), and cut-off frequency (*f*_*T*_) are analyzed in this section.

**Figure 9:**
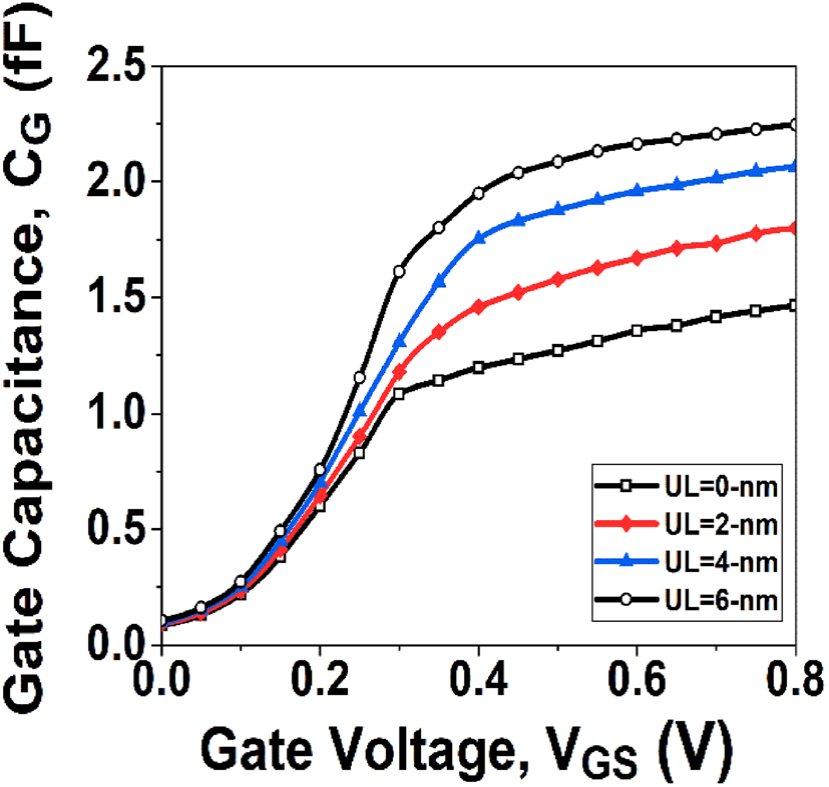
Gate capacitance (*C*_*G*_) with varying *V*_*GS*_.

The *C*_*G*_ of a device is an essential FOM for with RF applications. The *C*_*G*_ of a device can be calculated as the ratio between the change in charge carrier concentration and the change in *V*_*GS*_. The variation of *C*_*G*_ with respect to *V*_*GS*_ and underlap effect is shown in Fig. 9, and it is observed that with the introduction of underlap effect, the gate capacitance of the device rises. The peak value of *C*_*G*_ without UL mechanism is observed as 1.46 fF, whereas, with UL engineering of 6-nm, the maximum value of capacitance is 2.25 fF.

One of the crucial factors in determining a device’s RF performance is the cut-off frequency (*f*_*T*_). The frequency at which the current gain is equal to 0-dB is known as *f*_*T*_. The *f*_*T*_ is calculated by the following expression:

7 $${f}_{T}={g}_{m}/2\pi {C}_{G}$$

**Figure 10:**
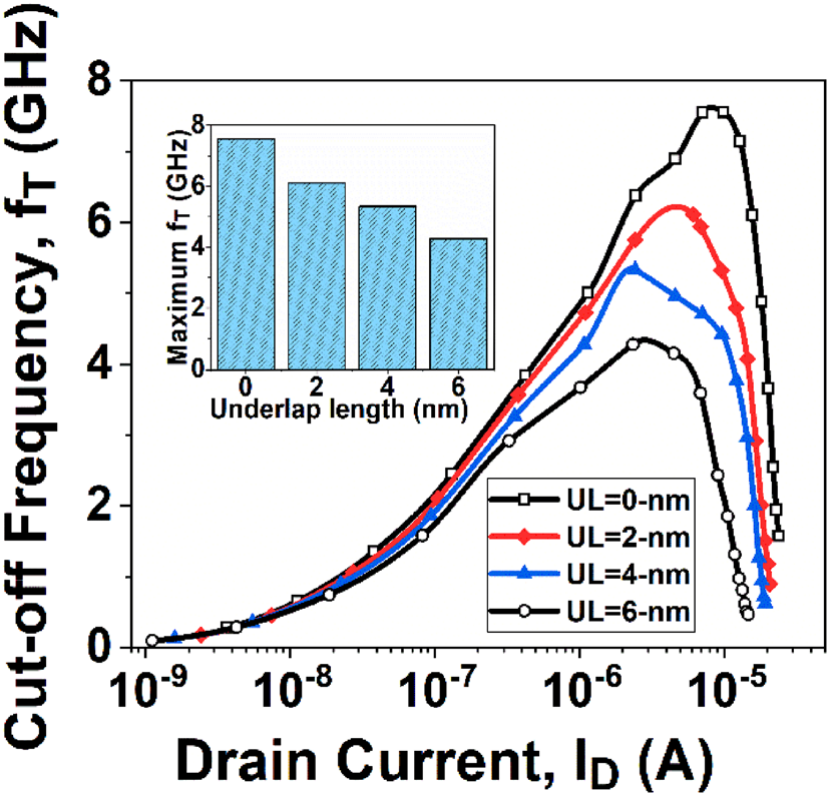
Cut-off frequency (*f*_*T*_) with varying *I*_*D*_.

Figure 10 shows the *f*_*T*_ variation with *I*_*D*_ for GNR-FET devices. According to Eq. (7), the *f*_*T*_ depends on *g*_*m*_ to *C*_*G*_ ratio; And the GNR-FETs with UL structure has a smaller value of *g*_*m*_ and a larger value of *C*_*G*_. Hence, it is obvious that *f*_*T*_ will decrease in devices with UL mechanism compared to a device with no underlap effect.

In the design of analog circuits, achieving a balance between device efficiency, bandwidth, and intrinsic gain is a critical factor. Trade-off analysis can be utilized to identify the optimal operating point by examining several metrics, including the gain frequency product (GFP) and gain transconductance frequency product (GTFP). GFP, which is calculated as GFP = (${g}_{m}{r}_{0}$)* f*_*T*_, is a significant property for operational amplifiers employed in high-frequency applications^40^.

**Figure 11:**
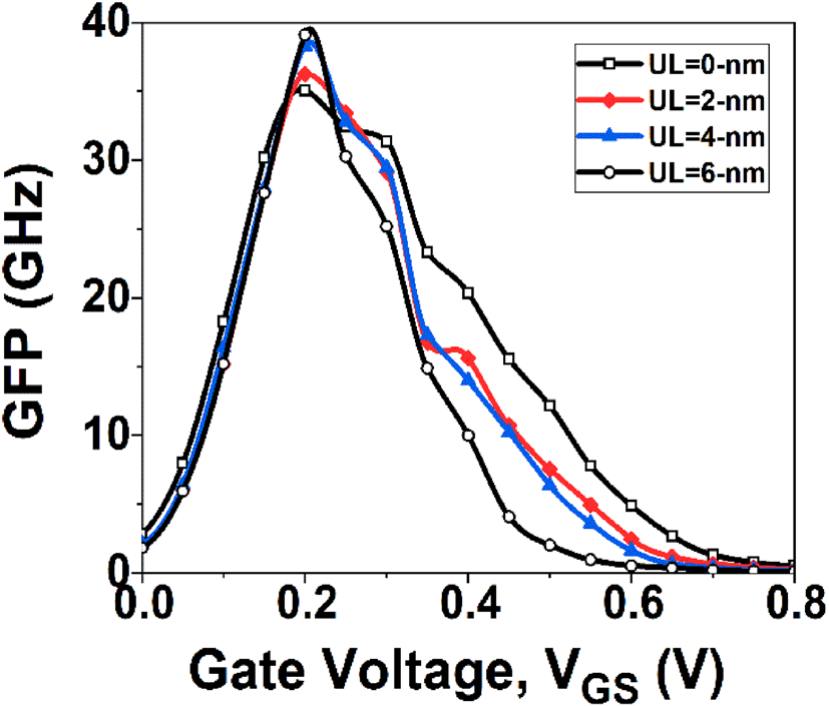
Plot of GFP with *V*_*GS*_.

**Figure 12:**
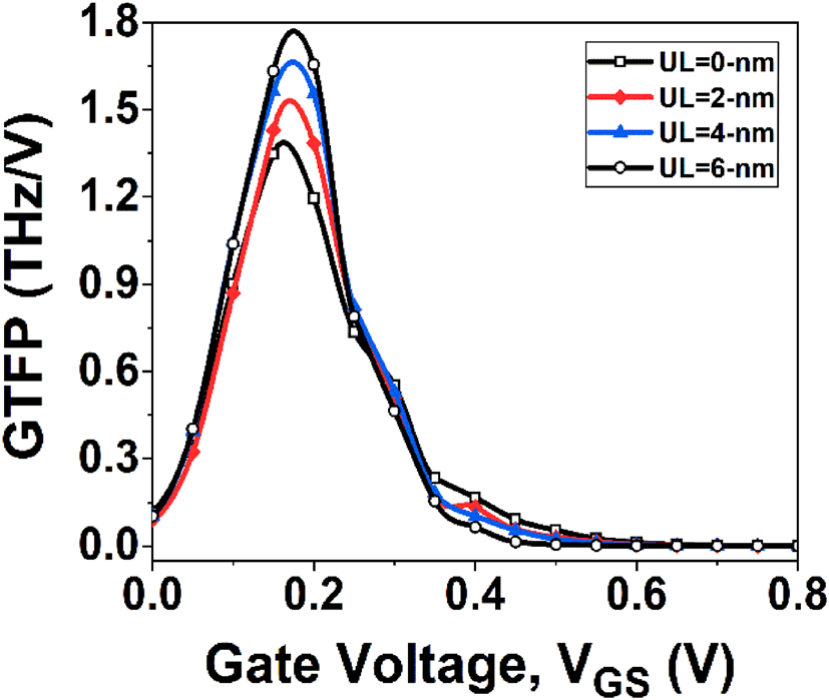
Plot of GTFP with *V*_*GS*_.

Figure 11 depicts GFP variation with *V*_*GS*_. The underlap effect produces the maximum value of GFP, while at low and high *V*_*GS*_, the GFP with no underlap effect has a higher value. However, to determine the best operating point for analog circuits, it is more critical to consider how device efficiency, inherent gain, and frequency can be traded off. As a result, GTFP is evaluated. A higher GTFP value enables the circuit designer to adjust gain, transconductance, and cut-off frequency to achieve the optimal operating region^41^. The GTFP is defined as the product of GFP and TGF. Figure 12 shows the variation of GTFP with *V*_*GS*_. It is observed that the GTFP value is highest for the GNR-FET device with underlap engineering, due to its higher transistor efficiency and output resistance.

### Impact of symmetric underlap length

In this section, we analyze the impact of symmetric underlap length on the transfer characteristics of the device and compare it with the optimized asymmetric Source/Drain extension. Specifically, we consider the optimized asymmetric underlap length as UL = 6-nm and a symmetric underlap of 6-nm for our comparison.

**Figure 13:**
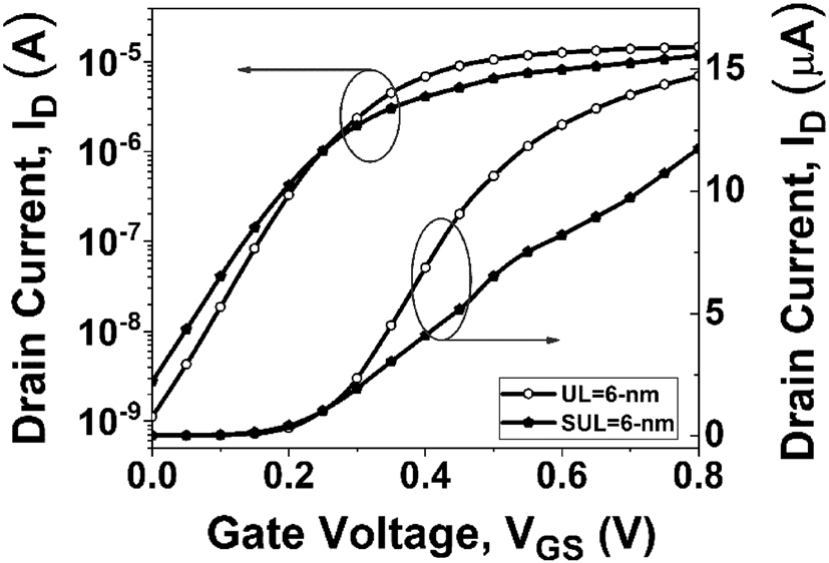
*I*_*D*_* -V*_*GS*_ of GNR-FETs with asymmetric and symmetric underlap length of 6-nm at *V*_*DS*_ = 0.3 V.

Figure 13 illustrates the impact of asymmetric underlap (UL) length and symmetric underlap (SUL) length on the *I*_*D*_ concerning the *V*_*GS*_ of GNR-FETs. It is evident from Fig. 13 that the drive current decreases, while the off-current increases considerably in SUL 6-nm compared to UL 6-nm underlap. This is due to the significant influence of series resistance in the region of operation. In a SUL DG FET, there exists a swapping between on-current and fringing capacitance. While utilizing the underlap engineering can decrease parasitic capacitance, it also results in higher source/drain resistances^30^. To make it viable for system-on-chip applications, where analog and digital circuits coexist on the same integrated circuit, efforts should be directed towards identifying the optimized device with the highest on–off current ratio.

### Linearity analysis

Linearity is a crucial requirement in RF applications^42^. To achieve a distortion-free output signal with minimal intermodulation and higher-order harmonics, MOS devices with high linearity are essential. Non-linearity in this context is typically associated with higher-order transconductance, representing higher-order derivatives of a transistor’s transfer characteristics. In this study, several metrics, namely *g*_*m2*_, *g*_*m3*_, VIP2 and IIP3^43^ are used to assess RF linearity of an asymmetric underlap length near the channel-drain junction, and compare it with the device without underlap.

We will begin by focusing on the impact of higher-order transconductance FOMs, specifically *g*_*m2*_ and *g*_*m3*_, which can introduce non-linearity by interfering with the fundamental frequency. To address this non-linearity, *g*_*m3*_ is considered the dominant parameter compared to *g*_*m2*_. The even-order harmonics in circuits can be effectively mitigated through balanced topologies, making the impact of *g*_*m2*_ manageable in maintaining high linearity. On the contrary, *g*_*m3*_ proves to be highly unpredictable, thus imposing lower limits on distortion. Consequently, minimizing the amplitudes of *g*_*m2*_ and *g*_*m3*_ to the greatest extent possible is crucial to achieving high linearity in RF applications.

The transconductance of the second order (*g*_*m2*_) and the transconductance of the third order (*g*_*m3*_). The *g*_*m2*_ and *g*_*m3*_ are determined as Ref.^44^:

8 $${g}_{m2} = \frac{\partial {g}_{m1}}{\partial {V}_{GS}}$$

9 $${g}_{m3} = \frac{\partial {g}_{m2}}{\partial {V}_{GS}}$$

**Figure 14:**
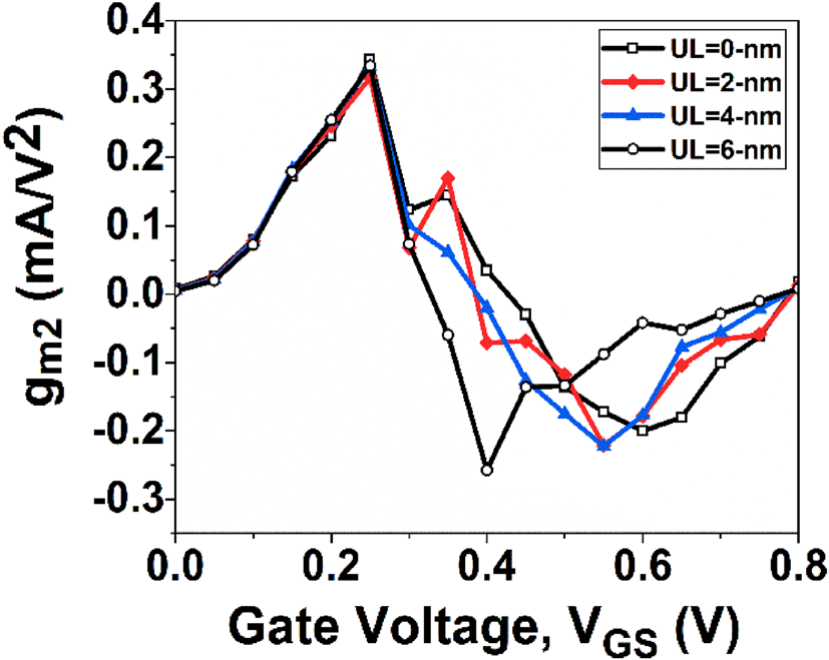
Variation of *g*_*m2*_ with *V*_*GS*_.

**Figure 15:**
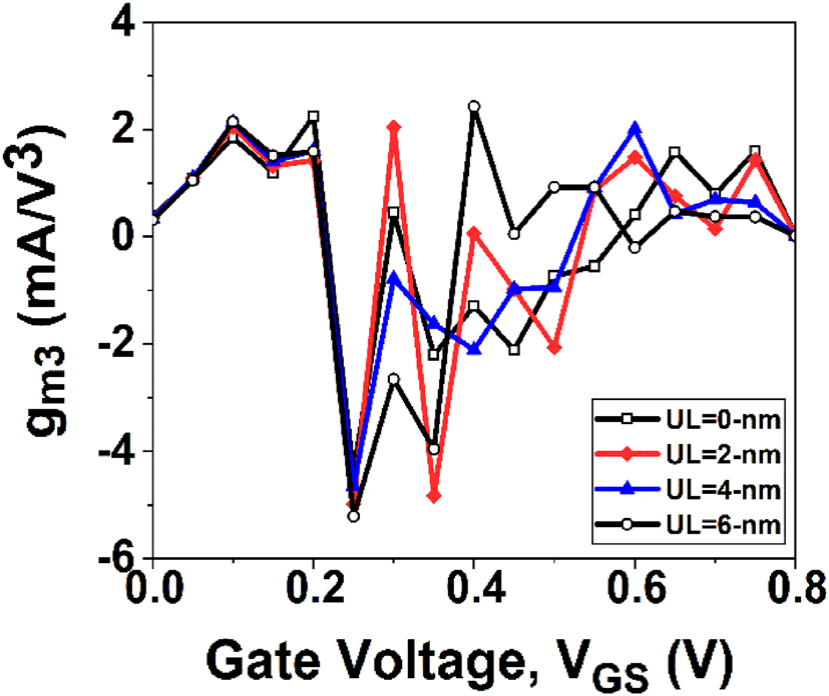
Variation of *g*_*m3*_ with *V*_*GS*_.

If a device has a greater peak value of *g*_*mn*_ at a lower *V*_*GS*_ compared to another device, it is considered to have better linearity^45^. Figure 14 displays the variation of *g*_*m2*_, while Fig. 15 depicts the variation of *g*_*m3*_ with *V*_*GS*_ for GNR-FET devices under study. It is interesting to note from Figs. 14 and 15 that conventional and UL GNR-FET devices have first peak values of *g*_*m2*_ and *g*_*m3*_ at the same *V*_*GS*_. Therefore, in order to determine the device with the best linearity among those under consideration, further investigation of linearity parameters such as VIP2 and IIP3 is required.

VIP2 is utilized to evaluate distortion characteristics based on dc parameters. Improved linearity performance and reduced distortion operation are attained with higher values of VIP2 and IIP3. The IIP3 represents the input power level at which extrapolation results in the first-order power being equal to the third-order power. Having a high IIP3 value allows for enhanced linearity performance operation. The VIP2 and IIP3 is given by Refs.^42,43^:

10 $$VIP2=4*\frac{{g}_{m1}}{{g}_{m3}}$$

11 $$IIP3=\frac{2}{3 {g}_{m3}}\frac{{g}_{m1}}{{R}_{S}}$$

where R_S_ represents source resistance. For most RF applications, R_S_ = 50 Ω is considered.

**Figure 16:**
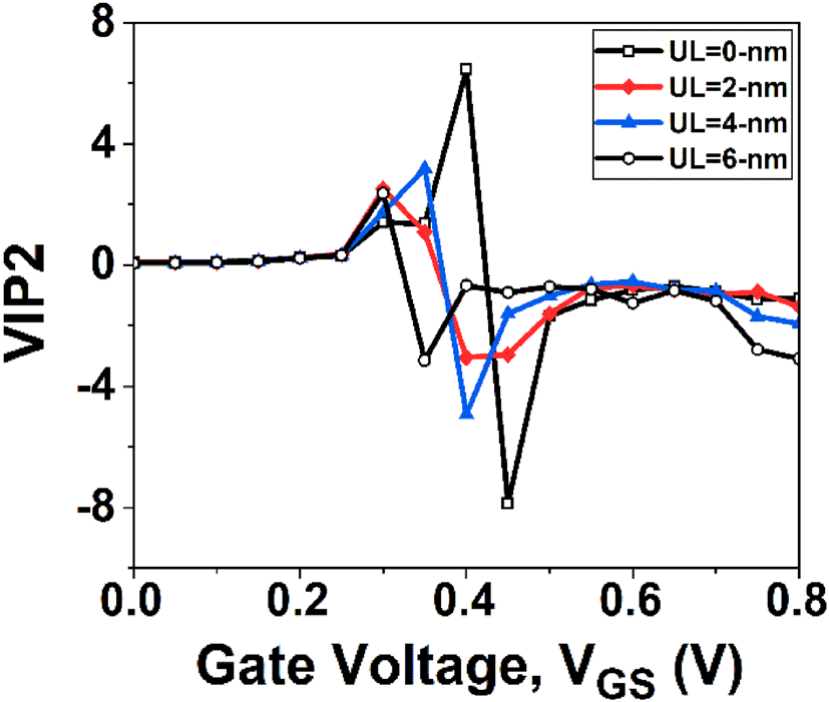
Plot of VIP2 with *V*_*GS*_.

**Figure 17:**
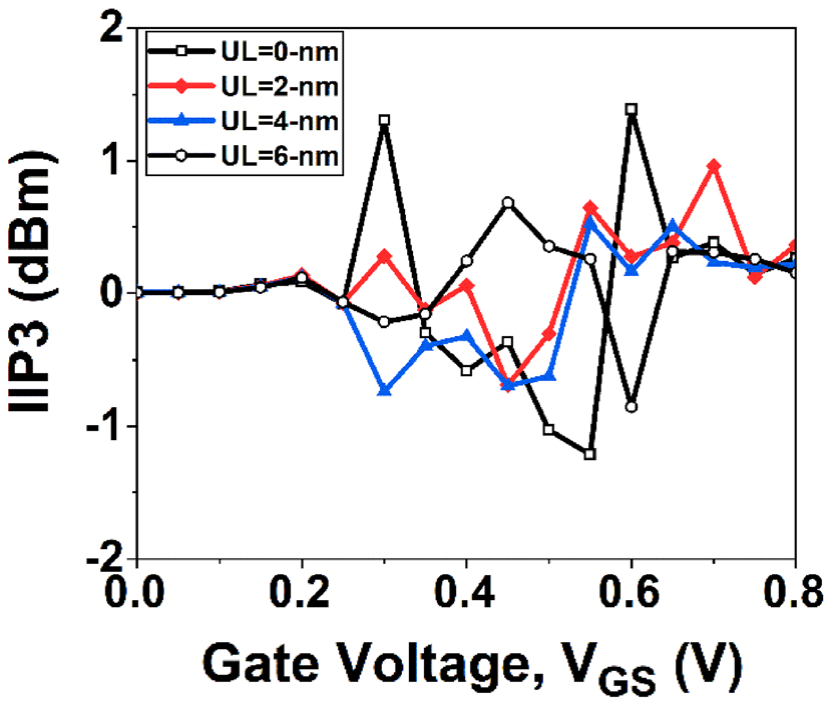
Plot of IIP3 with *V*_*GS*_.

The variation of VIP2 with *V*_*GS*_ is shown in Fig. 16. It can be observed from the figure that the device without underlap architecture exhibits a higher VIP2 value when compared to the UL design. Figure 17 shows the variation of IIP3 as a function of *V*_*GS*._ It is observed from Fig. 17 that the device without UL engineering has the maximum value of IIP3. Therefore, the GNR FET without UL structure is more linear in compared to the GNT FET devices with underlap architecture.

## Conclusion

In this study, the optimization of the underlap length and the comparative analysis of analog and RF FOMs for the GNR-FET device is performed. The study examines the impact of underlap structure on the drain side of the GNR channel in analog and RF applications, comparing it to an ideal device without underlap in the GNR channel. The optimized device with underlap structure demonstrates notable enhancements, including a 102% increase in *I*_*ON*_*/I*_*OFF*_ ratio and a 12.33% decrease in DIBL compared to the conventional GNR-FET device without underlap. Similarly, GNR-FETs with underlap structures exhibit a 38.49% increase in TGF and a 54.32% increase in intrinsic gain compared to conventional GNR-FET devices. The results also highlight significant changes in RF performance metrics, with a 53.41% increase in gate capacitance, a 11.48% increase in GFP, and an 22.78% increase in GTFP. However, the cut-off frequency of the GNR-FETs is reduced by 43.3% compared to the ideal GNR-FET device. Therefore, underlap engineering in GNR-FETs is particularly advantageous for analog circuit applications where high transistor efficiency (TGF), gain, GFP, and GTFP are of primary importance. This approach enables a balance of device efficiency, gain and frequency, making it well-suited for medium to high-frequency applications. However, for RF performance and stability, the device without underlap is preferable. Consequently, the discussed parameters exhibit high sensitivity to the underlap structure of GNR-FETs, and the underlap mechanism can be utilized to regulate the performance of double-gate GNR-FETs based on specific application requirements.

## Data Availability

The data that support the findings of this study are available from the corresponding author, [akram14407@gmail.com], upon reasonable request.
